# Nuclear Magnetic Resonance to Detect Rumen Metabolites Associated with Enteric Methane Emissions from Beef Cattle

**DOI:** 10.1038/s41598-020-62485-y

**Published:** 2020-03-27

**Authors:** R. Bica, J. Palarea-Albaladejo, W. Kew, D. Uhrin, D. Pacheco, A. Macrae, R. J. Dewhurst

**Affiliations:** 1 Scotland’s Rural College, SRUC, West Mains Rd, Edinburgh, EH9 3JG United Kingdom; 2 Royal (Dick) School of Veterinary Studies and the Roslin Institute, University of Edinburgh, Easter Bush Campus, Midlothian, EH25 9RG United Kingdom; 3 Biomathematics and Statistics Scotland, JCMB, Peter Guthrie Tait Road, The King’s Buildings, Edinburgh, EH9 3FD United Kingdom; 4 The University of Edinburgh, EaStCHEM School of Chemistry, The King’s Buildings, David Brewster Road, Edinburgh, EH9 3FJ United Kingdom; 5 AgResearch Grasslands Research Centre, Tennent Drive, 11 Dairy Farm Road, Palmerston North, 4442 New Zealand

**Keywords:** Animal physiology, Metabolomics

## Abstract

This study presents the application of metabolomics to evaluate changes in the rumen metabolites of beef cattle fed with three different diet types: forage-rich, mixed and concentrate-rich. Rumen fluid samples were analysed by ^1^H-NMR spectroscopy and the resulting spectra were used to characterise and compare metabolomic profiles between diet types and assess the potential for NMR metabolite signals to be used as proxies of methane emissions (CH_4_ in g/kg DMI). The dataset available consisted of 128 measurements taken from 4 experiments with CH_4_ measurements taken in respiration chambers. Predictive modelling of CH_4_ was conducted by partial least squares (PLS) regression, fitting calibration models either using metabolite signals only as predictors or using metabolite signals as well as other diet and animal covariates (DMI, ME, weight, BW^0.75^, DMI/BW^0.75^). Cross-validated R^2^ were 0.57 and 0.70 for the two models respectively. The cattle offered the concentrate-rich diet showed increases in alanine, valerate, propionate, glucose, tyrosine, proline and isoleucine. Lower methane yield was associated with the concentrate-rich diet (p < 0.001). The results provided new insight into the relationship between rumen metabolites, CH_4_ production and diets, as well as showing that metabolites alone have an acceptable association with the variation in CH_4_ production from beef cattle.

## Introduction

Livestock production is the largest anthropogenic contributor to the global CH_4_ budget (103 [95–109] Tg CH_4_ yr−1 during 2000–2009)^1^, with enteric CH_4_ emissions being the largest contributors to this (87–97 Tg CH_4_ yr−1 during 2000–2009)^1–3^. Methane is a potent greenhouse gas (GHG) with a global warming potential (GWP) 28 times higher than carbon dioxide (CO_2_), with a 12-year atmospheric lifetime^4^ and it contributes significantly to stratospheric ozone depletion^5^. Methane production is also associated with a net loss of energy to the animal, ranging from 2–12% of energy intake^6^. Methane production is mainly associated with fermentation of feed and occurs primarily in the rumen, with the rest (11% in one study) occurring in the lower hindgut^7^.

Rumen methanogens principally utilise H_2_ and CO_2_ to produce CH_4_ in the hydrogenotrophic pathway. Acetate and butyrate production results in more H_2_ being produced and available for CH_4_ production, whilst propionate results in less, as propionate production uses up H_2_, leaving less available for CH_4_ production^8,9^. Until recently, this was thought to be the sole pathway for CH_4_ production. However, another group of methanogens uses methyl-containing metabolites such as methanol and methylamine to produce CH_4_, utilising the methylotrophic methanogenic pathway^10^. This pathway is found in the *Thermoplasmata* genus of archaea showing enhanced growth when given methylamine supplements. The contribution of these substrates to CH_4_ production has not been assessed as yet, although it is known that these archaea can be stimulated by increasing dietary pectin concentrations, as well as increased grain content in the diet^8,11^.

The components of ruminant diets, especially carbohydrate type, heavily influence these pathways by altering the rumen microbiota. Many experiments have shown diet effects on CH_4_ production in ruminants^12^. These studies highlighted several important factors such as forage quality and forage to concentrate ratio affecting emissions. In general, a higher forage proportion in diets results in increased CH_4_ emissions. However, there is an ongoing requirement to assess the effects of different diets and supplements on CH_4_ emissions.

With increasing efforts to reduce the carbon footprint of ruminants, the need for an accurate and large-scale method to measure CH_4_ emissions at an individual animal level is necessary^9^. Current methods such as the sulfur hexafluoride (SF_6_) tracer method and respiration chambers (the latter seen as the ‘gold standard’ in the field) are accurate, but they are not suitable for large scale recordings on farm as they are expensive and low throughput. A recently published large review illustrates the potential proxies (indirect indicators/traits) including feed intake, milk composition and production, faeces, whole animal measurements and metabolites^13^. This review focused on describing, assessing and evaluating the suitability of each proxy in terms of accuracy, cost and potential. Recent research from our team has identified promise in the use of rumen metabolites as proxies for CH_4_ emissions^9,14^, and in the current study we extend the analysis by using nuclear magnetic resonance (NMR) spectroscopy with the aim of obtaining a more comprehensive description of the metabolites in rumen fluid. In the field of metabolomics there are 3 main analytical techniques used: NMR, gas chromatography coupled with mass spectrometry (GC-MS), and liquid chromatography coupled with mass spectrometry (LC-MS)^15^. Of these three, NMR is the least used as alternative techniques are regarded as more sensitive and with a greater detection potential. However, an increasing number of studies are utilising NMR^15,16^ which is why it was chosen for the current study.

The objectives of this work were to identify NMR signals and related metabolites associated with the different diets, with particular interest in metabolites belonging to the methylotrophic pathway, specifically methanol and methylamine, and assess the possibility of using these signals as a proxy for ruminant CH_4_ emissions.

## Materials and Methods

Each of the individual experiments in this study was approved by Scotland’s Rural College (SRUC) Animal Experiments Committee, which operates as the Local Ethical Review Group required under the UK Animals (Scientific Procedures) Act 1986. The studies were conducted at the SRUC Beef and Sheep Research Centre in Edinburgh, and all work was undertaken in accordance with the requirements of the UK Animals (Scientific Procedures) Act 1986.

### Animals and diets

A series of studies on CH_4_ emissions using respiration chambers (211 individual measurements) were conducted over a 3-year period (2013 to 2015). The cattle, which were a mixture of either steers or beef cows of varying breeds (Aberdeen Angus × Limousin; Limousin × Aberdeen Angus; Luing pure bred; Charolais cross bred), were bedded on wood fibre and sawdust to ensure that consumption of bedding would not contribute to nutrient intake. Water was offered *ad libitum* using water troughs, and diets were fed *ad libitum* once daily using electronic feeders (HOKO, Insentec, Marknesse, The Netherlands). Adaptation to the diet consisted of a 4-week period before entering the training pens. Before entering the respiration chambers, the cattle were housed in training pens, all identical in both size and shape to the chambers, for a period of one week. The cattle were subsequently moved to the chambers (each chamber having a volume of 76 m^3^) for a 72 h period where CH_4_ and H_2_ measurements were taken only in the final 48 h period. Chambers were ventilated by circulating fans set at 450 litres/s. Exhaust fans removed air at 50 litres/s, which approximated to 2.5 air changes/h. Methane production was calculated using results from an ADC MGA-300 multigas analyser, with the difference in inlet and outlet gas concentrations, multiplied by dry airflow, and corrected to atmospheric pressure and temperature (25 °C and 101,300 Pa). Two gas calibration procedures were taken prior to the start of the experiment: zero calibration to set all gas values to zero by passing nitrogen through them, and span calibration which passes gases of known concentrations (CH_4_, CO_2_, O_2_, H_2_) through the analyser. Daily gas production was calculated as an average of individual values and converted to a mass basis. The three diet types fed consisted of: (A) high concentrate diets (<100 g forage/kg DM) primarily comprising ground barley and barley straw with either distillers grain or rapeseed meal. (B) Mixed diets (400–600 g forage/kg DM) comprising either grass silage, barley, whole crop or barley and either distillers dark grain or rapeseed meal. (C) High forage diets (>700 g forage/kg DM) consisting of barley straw and either brewers grain or grass silage. Further details of the diets are provided in Table 1. It should be noted that only results from animals where we had full information on CH_4_, DMI and NMR analysis were used for the final analysis.

**Table 1:** Study number, chemical composition, and ingredients of diets split into high-concentrate (80.25 forage on average, g/kg DM), mixed (527 forage on average, g/kg DM), and high-forage (924.6 forage on average, g/kg DM) diets.

Study^1^	Diet*	Forage, g/kg DM	Starch,	NDF,	ME^2^,
			g/kg DM	g/kg DM	MJ/kg DM
Concentrate diet type (forage less than 100 g/kg DM)					
1	3	79	415	248	12.8
4	7	84	439	227	12.2
4	8	80	476	204	12
4	9	78	416	211	12.9
Mixed diet type (400–600 g forage/kg DM)					
1	4	505	284	374	12
4	10	490	298	289	11.6
4	11	499	318	272	11.4
4	12	497	262	280	12.2
2	14	557	281	308	11.6
2	15	558	308	295	11.4
2	16	555	264	317	11.9
2	17	556	247	313	11.6
Forage diet type (>700 g forage/kg DM)					
3	5	774	65	771	7.4
3	6	1000	0	693	8.1
5	13	1000	36	473	10.7

*Diet column referrers to the individual diets in each study with the corresponding numbers associating to a specific diet: 3–5 = straw, 4-6-7-10-14 = control, 8-11-15 = nitrate, 9–12 = rapeseed cake (lipid), 16 = maize dark grains (lipid) and 17 = nitrate + maize dark grains. ^1^Different studies used in the analysis please see methodology section. ^2^ME estimated from feed composition^46^.

Four of these studies have been previously published where full details of the methodology can be found^17–20^.

### Rumen sampling

Since rumen fluid sampling could not be done whilst the cattle were in the chambers, samples were taken from the animal shortly after it left the chamber (within a 2 hr time limit). A nasogastric tube (16 × 2700 mm Equivet Stomach Tube, Jørgen Kruuse A/S) was inserted through the nasal cavity and approximately 50 mL of rumen sample was obtained and filtered through 2 layers of muslin. The first part of samples were discarded if there was evidence of contamination by saliva or blood. Samples were immediately frozen at −20 °C until further analysis.

### Sample preparation and NMR data acquisition

Rumen samples were thawed 24 hours before use, centrifuged (2 mL; 13,000 rpm for 5 minutes) and filtered (Whatman 0.2 μm syringe filters)^11^. The supernatant was transferred into 2 mL tubes, and phosphate buffer (stock solution of 600 mM, pH 6.7) in D_2_O solution was added (50 μL) to 600 µL of rumen fluid samples, resulting in a final concentration of 50 mM. The spectral signals hardly shifted between samples, indicating the efficiency of the buffer. ^1^H-NMR spectra were generated on a 600 MHz Avance III (Bruker, Karlsruhe, Germany) spectrometer equipped with a 5 mm TCI Z-gradient pulsed-field gradient (PFG) cryoprobe. Spectra were acquired at 27 °C using noesygppr1d Bruker pulse program and the following parameters: 64 transients and 4 steady state scans using a 4.0 s and 2.7 s relaxation and acquisition time respectively. Water suppression (γB_1_ = 50 Hz) was applied during the relaxation delay and a 10 ms NOESY mixing time. A spectral width of 20 ppm was used for collecting 64 k data points. Pulsed field gradients (1 ms) were applied at the end of the pre-saturation (50%) and the mixing time (−10%). The spectra were acquired in an automated mode within 7.5 minutes of active data acquisition. The free induction decays (FIDs) were zero filled to 256 k data points and multiplied by an exponential weighting function corresponding to a line broadening (0.5 Hz) before Fourier transformation. Spectra were referenced to DSS (0.0 ppm, 1 mM) and manually corrected for phase and baseline distortions. The shift in spectral signals was negligible across samples. Manual binning was applied to integrate the area under the signal peaks using regions between 0.03 and 0.1 ppm to cover whole multiplets or overlapping multiplets, resulting in 128 integrals. Parts of the spectra containing water signal (4.4–5.2 ppm) and impurities such as glycerol were excluded. Integrals were calculated using the intser functionality of Topspin 3.5.

Three TOCSY (total correlation spectroscopy) experiments were acquired using DIPSI-2 and mixing times of 20, 40 and 60 ms, respectively. 2 ms sinc pulses were used for water suppression. The 2D ^1^H, ^13^C HSQC (heteronuclear single quantum correlation) -TOCSY used 25 ms DIPSI spin-lock. The 2D ^1^H, ^13^C HMBC used a two-state suppression of one-bond correlations set for ^1^*J*_CH_ = 125 and 160 Hz and long-range evolution interval optimised for ^n^*J*_CH_ = 8 Hz.

### Statistical analysis

#### Exploratory data analysis

The distribution of NMR signal integrals was graphically represented and compared using parallel boxplots. The data were subsequently processed by principal component analysis (PCA) to facilitate identification of patterns and interpretation. Loadings and scores from PCA were used to display the data set on a planar biplot based on the first two principal components (which accounted for 95% of the total variability), with points representing the samples and arrows from the origin indicating directions of increasing NMR signal integrals.

### Comparisons between concentrate and mixed diet types

Firstly, CH_4_ production (CH_4_ g/kg DMI expressed on a natural logarithm scale) was compared between concentrate and mixed diet types using a linear mixed model (LMM) fitted by restricted maximum likelihood (REML). This analysis was based on data from studies 1 and 4 (Table 1), for which information from these two types of diets was comparable as they used the same methodology. The model included diet type as a fixed effect, and study number as a random effect to account for variability between samples from both studies.

Furthermore, permutational multivariate analysis of variance (PERMANOVA)^21^ was used to compare the whole NMR spectral profiles between concentrate and mixed diet types.

### Predictive modelling

A predictive model of CH_4_ emissions (expressed on a natural logarithm scale) was fitted by partial least squares (PLS) regression using the kernel algorithm^22,23^. Firstly, only metabolite concentrations were considered as predictors. Then, the PLS model was re-fitted after including diet covariates (forage, starch, NDF in g/kg DM and ME (MJ/kg DM)) and animal covariates (DMI, ME, Weight, Weight^0.75^ and DMI/Weight^0.75^). The optimal number of PLS latent components was determined by 5-time repeated 10-fold cross validation aiming to minimize the root mean square error (RMSE) and maximize the coefficient of determination (R^2^) as model performance measures. The most parsimonious model amongst those reaching comparable highest performance following the one-standard error rule^24^ required either two latent components, when only metabolite concentrations were used as predictors, or four latent components, when diet and animal covariates were included as well. The prediction performance of the fitted PLS models was further evaluated using averaged R^2^ and RMSE from test data generated by 1000 random partitions of the data into calibration data (75% samples), used to tune and estimate the model, and test data (25% samples). The relative importance of the integrals as predictors of CH_4_ yield was assessed based on weights given to their model coefficients proportionally to reductions of the sums of squares across the number of PLS components. The top 20 most important integrals for each model were identified, and the ones corresponding to common non-VFA signals were used to produce a PCA biplot to explore their associations with diet types.

All the data analyses and modelling described above were conducted on the R system for statistical computing v3.5^25^. Statistical significance was assessed at the usual 5% significance level.

## Results

### Initial analysis of NMR spectra

Across all samples, signals related to common salts of volatile fatty acids (VFA)–acetate, propionate and butyrate–dominate. Additional minor signals in the alkyl, O-alkyl, carbohydrate and aromatic regions of the spectra are also present. A number of corresponding minor metabolites were identified through interpretation of 2D TOCSY, 2D ^1^H, ^13^C HSQC, 2D ^1^H, ^13^C HSQC-TOCSY and 2D ^1^H, ^13^C HMBC spectra of a representative sample and comparison with literature data^15,16^. The use of ^1^H-^13^C correlated experiments in this analysis was necessary as many ^1^H resonances overlap at 600 MHz and their identity in 1D ^1^H NMR spectra is obscured (the identified metabolites together with their ^1^H and ^13^C chemical shifts are summarised in Supplementary Table 1S). Assignment of metabolite resonances was also assisted by the analysis of the 1D sTOCSY spectra^15^ based on 128 rumen extracts spectra. In 1D sTOCSY spectra one “driver” peak is selected at the time and the R^2^ values are calculated across the spectral range and visualised by the colour of the peaks. While, as expected, the strongest correlations are observed between protons of the same compound, correlations between metabolites are also apparent.

Starting with the VFA, the colour coded median NMR spectra (Supplementary Figs. 1S–3S) highlight the intra- and, importantly, the inter-molecular correlations between metabolites showing similar concentration trends. Inspection of these spectra indicated that (i) acetate and butyrate are highly correlated, (ii) propionate correlates more strongly with acetate than with butyrate, and (iii) in general, correlations of VFA with other minor metabolites increased in order of butyrate < acetate < propionate. These correlations, and also correlations between minor metabolites, are presented later in the text.

### Exploratory data analysis

**Figure 1:**
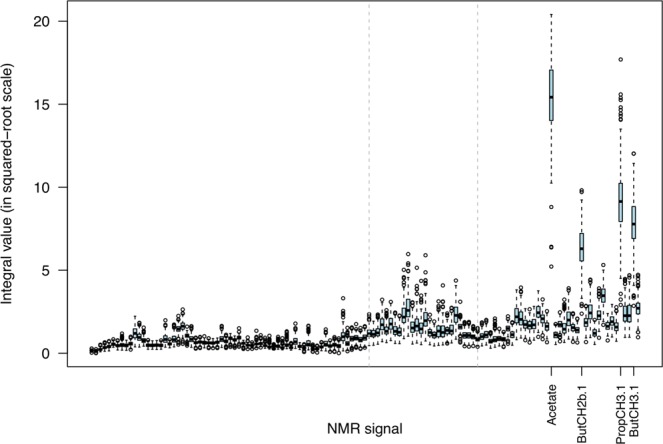
Distribution of rumen NMR signal integrals. Highlighted middle section (between dashed lines) corresponding to signals which, along with VFAs, showed association with methane yield. Data summarising the 128 metabolites across all the 211 samples.

To provide a visualisation of the distribution of signals present in all samples, the integral intensities were square-root transformed and are shown in Fig. 1. In this representation, signals related to common VFA (acetate, propionate and butyrate) appear as the most abundant across all samples. Some variability is apparent in levels of non-VFA metabolites in the O-alkyl/carbohydrate part of the spectra (3.2–4.2 ppm section between dashed lines in Fig. 1).

**Figure 2:**
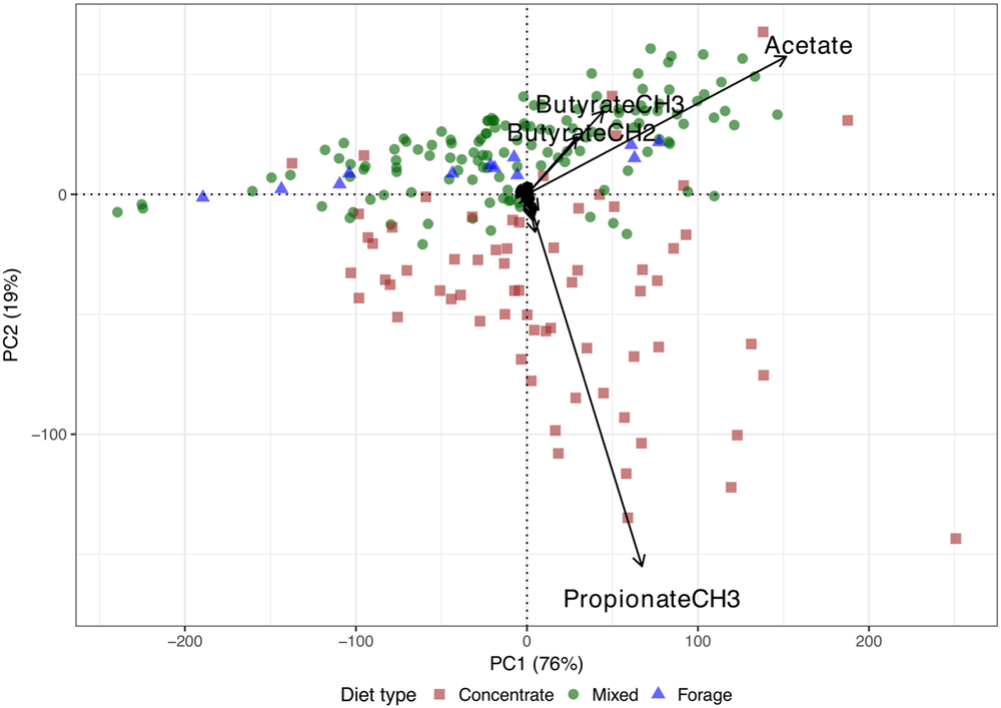
PCA biplot of rumen ^1^H NMR spectral data. Samples (points) distinguished by diet type and main VFA signals (arrows) labelled. The first principal component (PC1) is primarily associated with predominance of acetate along with butyrate, and the second principal component (PC2) primarily associated with predominance of propionate.

The data set was effectively represented by a PCA biplot based on the first two principal components (Fig. 2), which accounted for about 95% of the total data variability (76% explained by the first principal component, PC1, and 19% explained by the second principal component, PC2). According to the configuration of the biplot rays, values in PC1 were mostly representing variation in acetate, with points to the right of the origin associated with above-average levels for all VFA. Values in PC2 were mostly driven by propionate content, essentially separating samples according to the comparison between propionate and butyrate species.

Because of the low levels of the other signals, these were represented by fairly short vectors on the scale of the biplot, and they were hardly distinguishable (only main VFA were labelled to facilitate visualisation). The samples (points) were differentiated by symbols and colours according to the three main diet types (concentrate, mixed and forage). The angle between biplot rays is proportional to the correlation between the respective signals. Thus, acetate and butyrate species were more closely correlated, and mostly associated with mixed and forage diets, whilst propionate was present primarily in concentrate diet samples. The weak correlation between the acetate-butyrate axes and the propionate axis is demonstrated by the approximately 90-degree angle between them and is in accord with initial analysis of the 1D sTOCSY spectra presented above.

### Comparisons between diets

**Figure 3:**
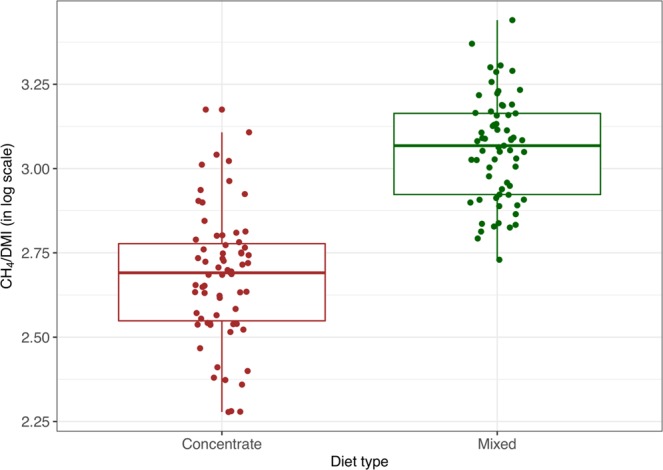
Boxplots displaying the distribution of CH_4_ emissions (on log scale) for concentrate and mixed diet type samples. Statistically significant difference in means was concluded from a linear mixed model (p < 0.001).

As observed in Fig. 3, which displays the actual observations along with their corresponding boxplots, the samples from mixed diet tended to produce higher levels of methane emissions (CH_4_ g/kg DMI expressed in a log scale) when compared with samples from concentrate diet. The difference was statistically significant according to LMM estimates (p < 0.001). Predicted marginal means and associated 95% confidence intervals were calculated from the LMM fit for each diet type and are summarised in Table 2.

**Table 2:** Predicted marginal means (PMM), standard errors (SE) and 95% confidence intervals (CI; lower and upper limits) from linear mixed model fitted to CH_4_ yield (in log scale) by diet type, using data from experiments 1 and 4.

Diet	PMM	SE	Lower CI limit	Upper CI limit
Concentrate	2.678	0.042	2.143	3.213
Mixed	3.056	0.042	2.519	3.593

In addition to this, the entire NMR signal profiles were compared between concentrate and mixed diet types using PERMANOVA and statistically significance differences in mean between them were noted (p = 0.001).

### Prediction of methane production from rumen NMR signals

Two PLS regression models were fitted to CH_4_ production (CH_4_ g/kg DMI in log scale), one including only NMR signals as predictors, and another one including these along with diet and animal covariates. Table 3 summarises model performance measures. The correlations (*r*) between observed and predicted CH_4_ g/kg DMI values (in log-scale) were 0.75 and 0.84 respectively for each model (graphs of observed against predicted values can be found in the Supplementary Fig. 13S). The table also presents R^2^ and RMSE measures based on cross-validated and test data, which show comparable results implying an improvement in R^2^ of around 20% and a reduction in RMSE of around 15% when covariates are added along with the NMR signals. Note that applying Pareto scaling on the predictors did not improve model performance and increased the number of latent components used.

**Table 3:** PLS predictive model performance using solely NMR signal integrals as predictors and adding diet and animal covariates.

Model	*r*	R^2^_cv_	RMSE_cv_	R^2^_t_	RMSE_t_
NMR integrals only	0.7517	0.5717	0.1626	0.5655	0.1642
NMR integrals + covariates	0.8382	0.7004	0.1365	0.6817	0.1403

*r*: Pearson’s correlation coefficient between predicted and observed values; R^2^: coefficient of determination (cross-validated, CV, and test data-based, t); RMSE: root mean squared error (cross-validated, CV, and test data-based, t).

**Figure 4:**
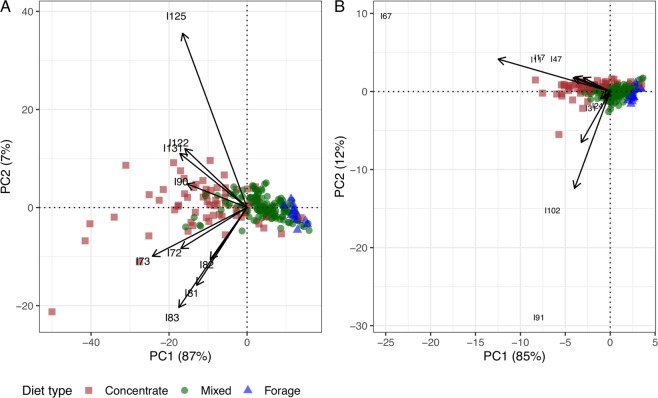
(**A**) PCA biplot based on common non-VFA signals amongst the top 20 most important signals for methane yield prediction derived from PLS modelling results. (**B**) PCA biplot of other NMR signals showing strong correlations with the top 20 signals. Samples distinguished by diet type. The correspondence of integral IDs with individual metabolites is given in the text.

The top 20 NMR signals in terms of predictive ability were extracted for both models (the corresponding PLS regression coefficients are indicated in Supplementary Fig. 13S), and the associated metabolites were identified using the sTOCSY-HSQC method described previously. The top 20 predictors in both models were mostly present in the aliphatic and carbohydrate region of the spectra and these included glucose (I72-72, I81–82–83), valerate (I125), tyrosine, lysine and putrescine (overlapping signals I90), alanine (I122) and valine, leucine and isoleucine (overlapping signals at I131). The associations between these non-VFA signals in common between both top 20 lists and diet type were investigated using a PCA biplot (Fig. 4A). Additionally, Fig. 4B displays the results of the PCA biplot analysis based on other NMR signals that showed strong correlations with both VFA and non-VFA signals amongst the top-20 in order to explore them further. The signals included hypoxanthine (I11), uracil (I17 and I47), tyrosine (I24 and I31), proline (I67) and 3-phenylpropionate (I91 and I102). Similar to the analysis considering all ^1^H NMR signals (Fig. 2), analysis of NMR minor signals separated the concentrate from the mixed/forage diets. The most significant metabolites identified in this analysis were glucose and amino acids, which were all positively correlated with the concentrate diet as opposed to the mixed/forage diets. The concentration of amino acids correlated highly with each other (I90, I122, I131), and to some extent with glucose (I72, I73 and I81, I82 and I83). As glucose signals appear in the most overlapped regions of the spectra, correlation of I72-73 with I81–83 integrals was seen due to contribution of other signals present in these regions. With regards to the metabolites identified that were correlated with the top 20 metabolites of both models, hypoxanthine and uracil were highly correlated to each other, and to a lesser extent to proline. All were positively associated with the concentrate diet. This is the contrary to what is noted with tyrosine and 3-phenylpropionate which seemed to be more associated to the mixed diet, particularly in the case of 3-phenylpropionate (Fig. 4B).

1D sTOCSY in addition identified several other metabolites whose signals were not amongst the top 20 ranking integrals, namely phenylalanine, aspartate, phenylacetate, xanthine, methanol and methylamine (Supplementary Table 1S). Inspection of 1D sTOCSY spectra of hypoxanthine and uracil (Supplementary Figs. 7S–8S) confirmed strong correlation of these signals with both glucose and amino acids while 3-phenylpropionate and valerate (Supplementary Figs. 9S-10S) showed weaker correlation with these metabolites. Phenylacetate, phenylalanine and aspartate exhibited strong correlations with glucose, most of the amino acids identified (alanine, lysine, leucine) hypoxanthine and uracil (Supplementary Figs. 4S-8S), indicating their link to the concentrate diet. However, it was noted that when using 3-phenylpropionate and valerate as driver peaks, this correlation was not observed.

While the first two VFA, acetate and butyrate, do not correlate with glucose, amino acids, hypoxanthine and uracil, propionate shows correlations with these metabolites (Supplementary Figs. 2S, 3S and 4S–8S). As to the identified metabolites which were related to the methylotrophic pathway, methanol and methylamine (Supplementary Figs. 11S and 12S), both were present at low concentrations throughout the spectra. When using methanol as a driver peak it was observed that it was more associated to acetate and propionate than butyrate. For methylamine, a higher association was noted with propionate compared to both acetate and butyrate. Both these metabolites showed a link to the metabolites which were associated with the concentrate diet.

## Discussion

The present study used NMR to identify rumen metabolites associated with CH_4_ emissions from beef cattle. The primary objectives of this study were to assess NMR as a metabolomics tool, determine whether the metabolites associated with the methylotrophic pathway could be increased with a diet with a high concentrate percentage, determine the effects of diet on CH_4_ emissions, and assess the predictive abilities of these NMR metabolite signals. The results obtained in this study helped us achieve our objectives, either in part or fully, in the following manner.

With regards to the effects of diet on CH_4_ emissions, there was a clear difference in CH_4_ emissions yield (g/kg DMI), with the concentrate diet being associated with the lowest CH_4_ emissions, and the mixed with the highest (Fig. 3). The concentrate diets had higher levels of starch compared to the mixed diet, and were composed of ground barley and barley straw compared to barley and grass silage of the mixed diets. A concentrate diet is described as being high in nutrient content and low in plant fibre^26^, and the CH_4_-reducing effect that concentrate diets have can be described in two ways: proportion of concentrate and/or composition of concentrate. Composition of concentrate effects were described in a study which showed that by increasing the starch content in the diet there would be an increase in starch fermenting microbes, leading to a shift in VFA production from acetate to propionate, and therefore a reduction in CH_4_ emissions^27^. An increased starch content in the diet increases propionate production by creating an alternative H_2_ sink^28^, lowers the pH of the rumen and ultimately inhibits the growth of methanogens^29^. With regards to proportion of concentrate in the diet, it has been shown that diets containing around 30% to 40% concentrate proportion have a relatively constant CH_4_ inhibition, whereas diets containing between 80% to 90% show elevated CH_4_ inhibition^30,31^. Thus, concentrate diets with high proportions of starch and barley lead to lower CH_4_ emissions, which was observed in the current study with CH_4_ emissions being lower for the concentrate diet.

Once we had established the relationship between the different diets and CH_4_ emissions,we observed how VFA concentrations varied. The primary observation was that, as expected, acetate and butyrate were mostly associated with the mixed diet (higher CH_4_) whereas propionate was mostly associated with the concentrate diet (lower CH_4_) (Fig. 2). This relationship is well-documented in the literature^6,32^. The VFA which were associated with H_2_ production, acetate and butyrate, were related to each other and had a clear distinction from the VFA which are associated with H_2_ consumption, namely propionate and valerate^9^. This can be explained because forage diets are characterised by large amounts of structural carbohydrates, and concentrate diets are characterised by low structural carbohydrates and high levels of starch^33^. However, what is interesting in this current study are the smaller signals, of which a total of 29 were identified (Supplementary Table 1S). Previous research has shown that a diet with readily degradable carbohydrates is associated with an increase in VFA concentrations in the rumen and a greater change to the rumen environment^34^. The concentrations of these can vary within the rumen due to passage rate, feed type, pH and H_2_ level. The current study confirmed this, with the most significant metabolites identified, after the main VFA, being sugars (mainly glucose) and amino acids. The amino acids identified included valine, leucine, isoleucine, tyrosine, lysine and proline. The increase of these amino acids is attributed to the greater availability of glucose, being a major monosaccharide which is liberated following an increased degradation of starch in the concentrate diet^11^, allowing it to convert to a large amount of polyols and amino acids via the glycotic pathway^35^. Hence, the amino acids related with glucose were also linked to the concentrate diets (Fig. 4A,B). This is in line with what is seen in a study in which a hay:concentrate diet was administered with low, mid and high levels of chloroform-cyclodextrin (a CH_4_ inhibitor), resulting in a linear increase in amino acids (isoleucine, valine, proline), as well as an increase in sugars ribose and arabitol^36^. Another cluster of metabolites which was associated with the concentrate diet included xanthine, hypoxanthine, alanine and uracil. Hypoxanthine, xanthine and uracil are degradation products of rumen bacteria, and alanine is released following the death of both Gram-positive and Gram-negative bacteria^11^. The well-established relationship with concentrate diets reducing rumen pH explains the increased concentration of alanine seen in this current study^37^. Uracil and hypoxanthine were shown to increase following the degradation of DNA/RNA when added to the rumen^38^. This relationship was also noted by Saleem *et al*.^39^, and may be indicative of a high level of bacterial cell lysis and subsequently a large change in the rumen microflora. A positive correlation was noted between this cluster and the amino acids mentioned above, indicating that they may be used as biomarkers of concentrate diets. In the region of the spectra in which glucose is present (3.2–3.8 - observation made through the Human Metabolome Database HMDB)^40^, the identification of metabolites was more complicated due to the overlapping of several metabolites. However, associations were observed between some metabolites (NMR integral I72/73-I81–82) and at the diet level (positive association with the concentrate diet). Despite the fact that many metabolites seem to be highly correlated to the concentrate diet, there are metabolites which decrease with concentrate proportion, most notably 3-phenylpropionate. Increasing glucose levels has shown negative effects on the concentration of 3-phenylpropionate, a relationship which was also noted by Turlin *et al*.^41^, indicating how glucose strongly represses the expression of genes which code for key enzymes in the 3-phenylpropionate production pathway. The increase in pH caused by the forage based diets^11^ also led to an increase in 3-phenylpropionate, as well as other organic acids, notably acetate. It is also interesting to note the changes in 3-phenylpropionate due to its abundance in the aromatic region of a spectra (7–9 ppm). Previous work has shown that 3-phenylpropionate and phenylacetate make up 64.3% (50.8% and 13.5% respectively) of the entire aromatic area of an NMR spectra in rumen fluid from animals consuming high-forage diets^11,39^. Thus, by shifting to a predominantly concentrate based diet, it is possible to modify this ratio and further explore the aromatic region within an NMR spectra. With regards to metabolites associated with the methylotrophic methanogens identified in the study, methanol seemed to be less associated to the concentrate diet when compared to methylamine (Supplementry Figs. 11S and 12S). It was also more associated with acetate when compared to methylamine. This agrees with results of an *in vitro* study which cultivated different mediums with methanol with presence and absence of H_2_. The medium cultured with methanol alone showed little methanogensis when compared to when H_2_ was present^42^. The fact that methanol and methylamine are seemingly not correlated may arise from the fact that methanol is only used by methanogenic archaea *Methanosphaera spp*^26^. Methylamine on the other hand exhibited characteristics observed in previous studies^11,35,39^, where it was more associated to propionate and therefore more linked to the concentrate diet, as observed in the current study. However, in the studies cited, a significant difference in methylamine was only noted at very high rates of barley grain in the diet^11,39^, whereas in the study by Zhao *et al*.^35^, a difference in methylamine concentrations was not observed when comparing diets (corn stover vs a mixture of alfalfa hay, *Leymus* chinensis hay and corn silage) but when comparing before and after feeding with both diets. A similar study^43^ looking at 3 diets (corn silage, grass silage and grass hay) showed that methylamine was less abundant in the corn silage diet, with highest concentrations exhibited in the grass silage diet. Ultimately suggesting how methylotrophic methanogenic activity may be greater in fiber rich diets. The *Thermoplasmata* genus has also been shown to utilise methylamine, as well as trimethylamine, as a source of energy and carbon, and these derive from the degradation of betaine, choline and methanol^10^ which could be the explanation as to why methylamine is more abundant in grass-based diets when compared to corn based diets^43^. The lack of consistency in results here and in the literature suggests that more research is required.

In the current study linear mixed modelling was useful to identify which diet related to greater CH_4_ emissions. The associations of rumen metabolites with CH_4_ emissions and diet types were investigated through multivariate statistical analysis using PCA, PERMANOVA and PLS regression. The exploratory data analysis showed interesting associations between the main VFA (acetate, propionate and butyrate) and diet (Fig. 2), while also highlighting the fact that the majority of the variation in CH_4_ can be described by the three main VFA. Studies have been undertaken to try and model different variables which might be correlated to CH_4_ emissions with varying results. Auffret *et al*.^14^ aimed to identify robust microbial biomarkers which could be used to predict CH_4_ emissions. By applying a simple regression analysis they identified the archaea:bacteria ratio as a biomarker for methane emissions, and a PLS analysis (which included 56 genera, diet effects and breed types) explained 50% of variation in methane yield. The results of a similar study which was looking at non-VFA related metabolites in milk, identified with NMR, and methane yield (g/kg DMI) were not far from those obtained in the current study, with their model providing R^2^ equal to 0.41 and *r* equal to 0.69^44^. Overall, what can be noted from the literature is that when trying to predict CH_4_ emissions with metabolite-based models, goodness of fit as measured by the R^2^ coefficient is generally between 0.3–0.5. Therefore, comparing our current results with these studies, even if comparison based on the R^2^ measure only is a notable simplification as it depends on technical details of the modeling used and particuliarities of the data in each study, we find that they are in line with previous work^11,14,44,45^, although ours were derived from solely utilising NMR metabolic profiles as predictors. It should also be noted that, similarly to the studies mentioned above, predictive abilities improved following addition of both animal and diet covariates (R^2^ = 0.70), reinforcing the evidence that these factors are still the main influencers on CH_4_ emissions.

The metabolites which were responsible for the predictive ability of our models described above, not including the VFA, were also very similar to the study by Saleem *et al*.^39^, which concluded that the top 10 metabolites which were responsible for explaining the difference between the 15% to 45% barley grain diet included putrescine, aspartate, alanine, glucose, methylamine, ferulate and formate. These metabolites are similar to the ones identified in our study with glucose, alanine and putrescine all being present in the top 20 identified (Fig. 4A). The differences in the top predictors between diets could be attributed to the fact this difference may be noted when production rates are very high^11,39^.

This study shows the potential to use metabolites as predictors of CH_4_ emissions in beef cattle, contributes to build knowledge on the use of NMR as a tool to obtain, quantify and identify rumen metabolites and provides valuable insight on how different diet types alter the rumen environment at a metabolic level. Future studies in this field would benefit from a larger metabolite database for identification which would allow a greater understanding of which metabolites are directly associated with CH_4_ production pathways in the rumen, and therefore improve the use of metabolites as a predictive tool for CH_4_ emission.

## Supplementary information

Supplementary Information.
