# Supplementary material for: Nuclear Magnetic Resonance to Detect Rumen Metabolites Associated with Enteric Methane Emissions from Beef Cattle
Source: Sci Rep. 2020 Mar 27;10:5578. doi: 10.1038/s41598-020-62485-y (PMC7101347; doi:10.1038/s41598-020-62485-y)
Supplement: Supplementary file 1 — Supplementary Information. [file 41598_2020_62485_MOESM1_ESM.pdf]

Supplementary Information for

Nuclear Magnetic Resonance to Detect Rumen Metabolites Associated with Enteric Methane Emissions from Beef Cattle

R.Bica[1,2*], J.Palarea-Albaladejo[3], W. Kew[4], D.Uhrin[4], D. Pacheco[5], A.Macrae[2], R.J.Dewhurst[1]

Riccardo Bica
Email: Riccardo.bica@sruc.ac.uk

This PDF file includes:

Supplementary Figures 1S-13S
Supplementary Table 1S

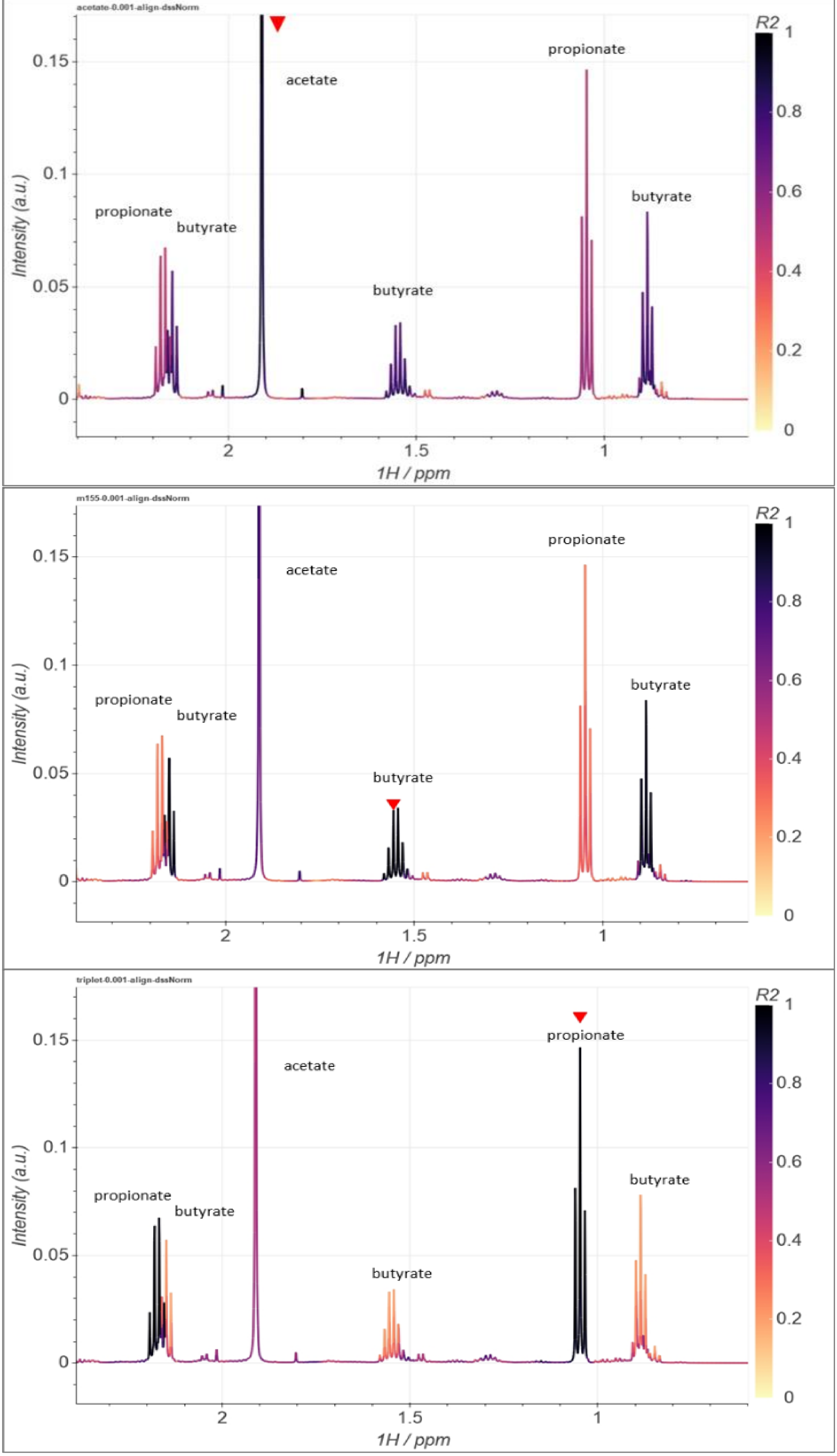

Supplementary Figure 1S. Colour coded median 1D ^{1}H spectra obtained by using the signal of **acetate** at 1.91 ppm (top left), **butyrate** at 1.55 ppm (top right) and **propionate** at 1.04 ppm (bottom left) as the driver signal. The square of the Pearson correlation coefficient, R^2, is coded into the colour of the peaks. Region 0.6 to 2.4 ppm is shown.

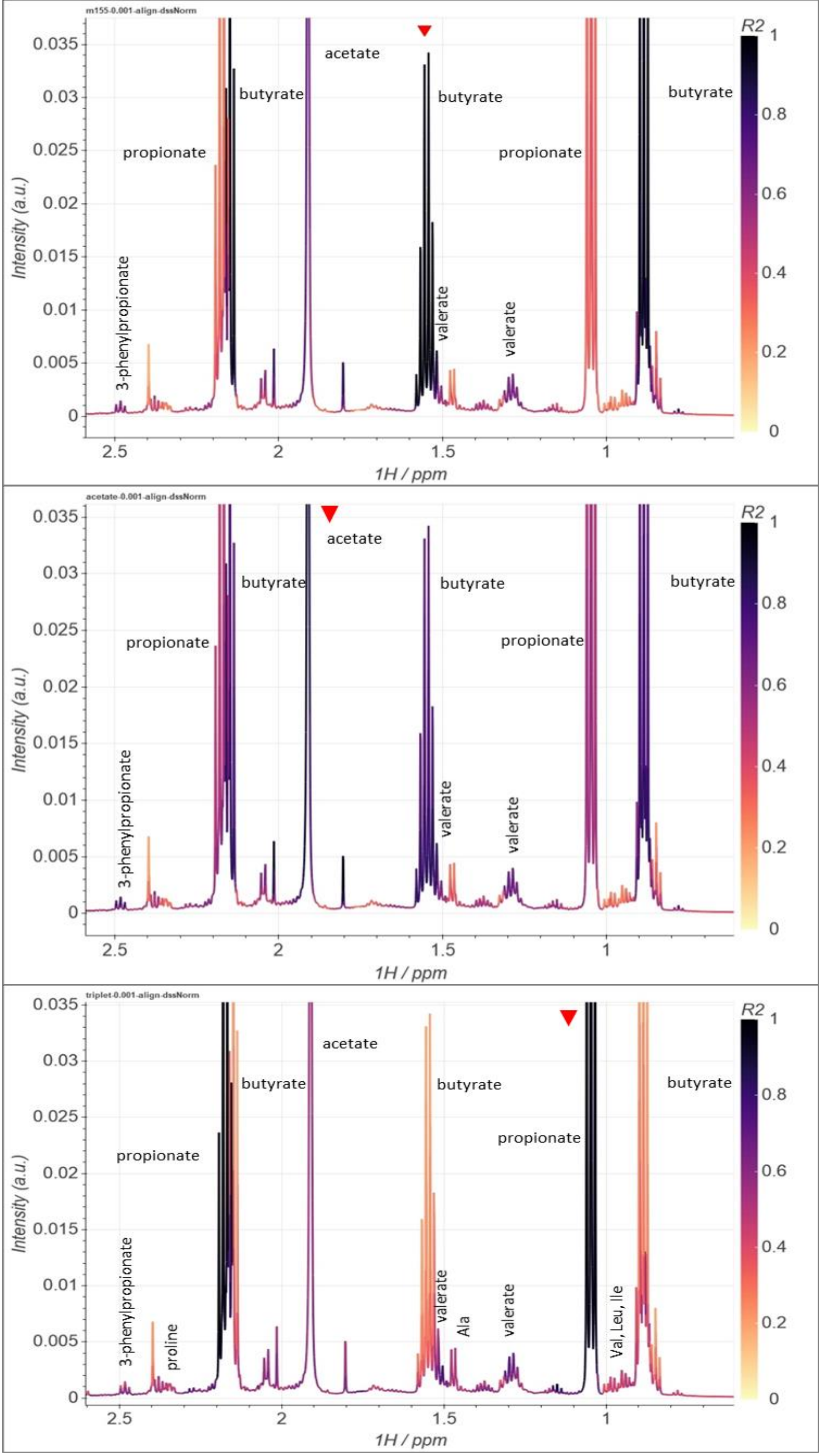

Supplementary Figure 2S. The same as Figure 1S, but the vertical scale was increased to visualise minor metabolites.

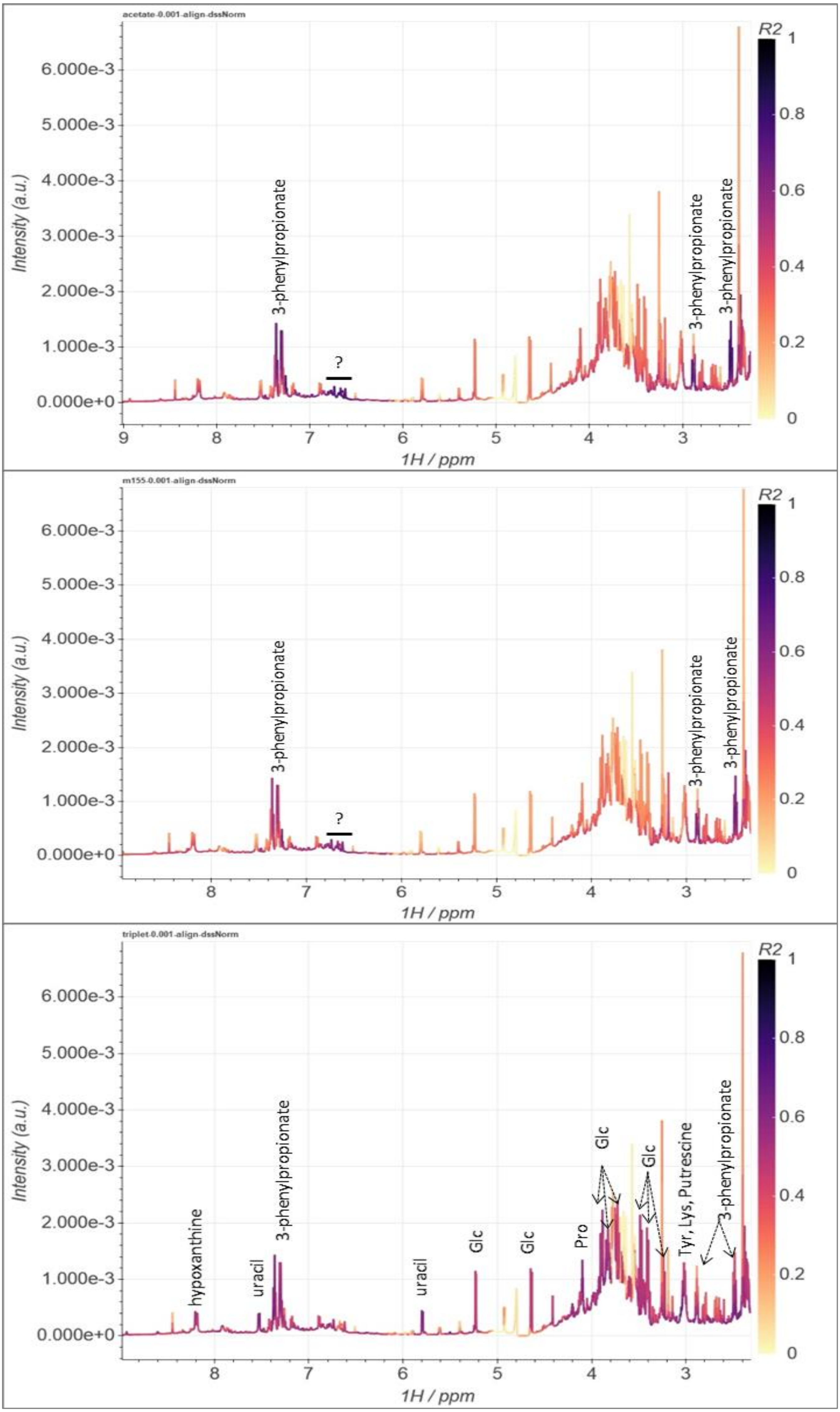

Supplementary Figure 3S. Colour coded median 1D ^{1}H spectra obtained by using the signal of **acetate** at 1.91 ppm (top), **butyrate** at 1.55 ppm (middle) and **propionate** at 1.04 ppm (bottom) as the driver signal. The square of the Pearson correlation coefficient, R^2, is coded into the colour of the peaks. Region 2.4 to 9 ppm is shown.

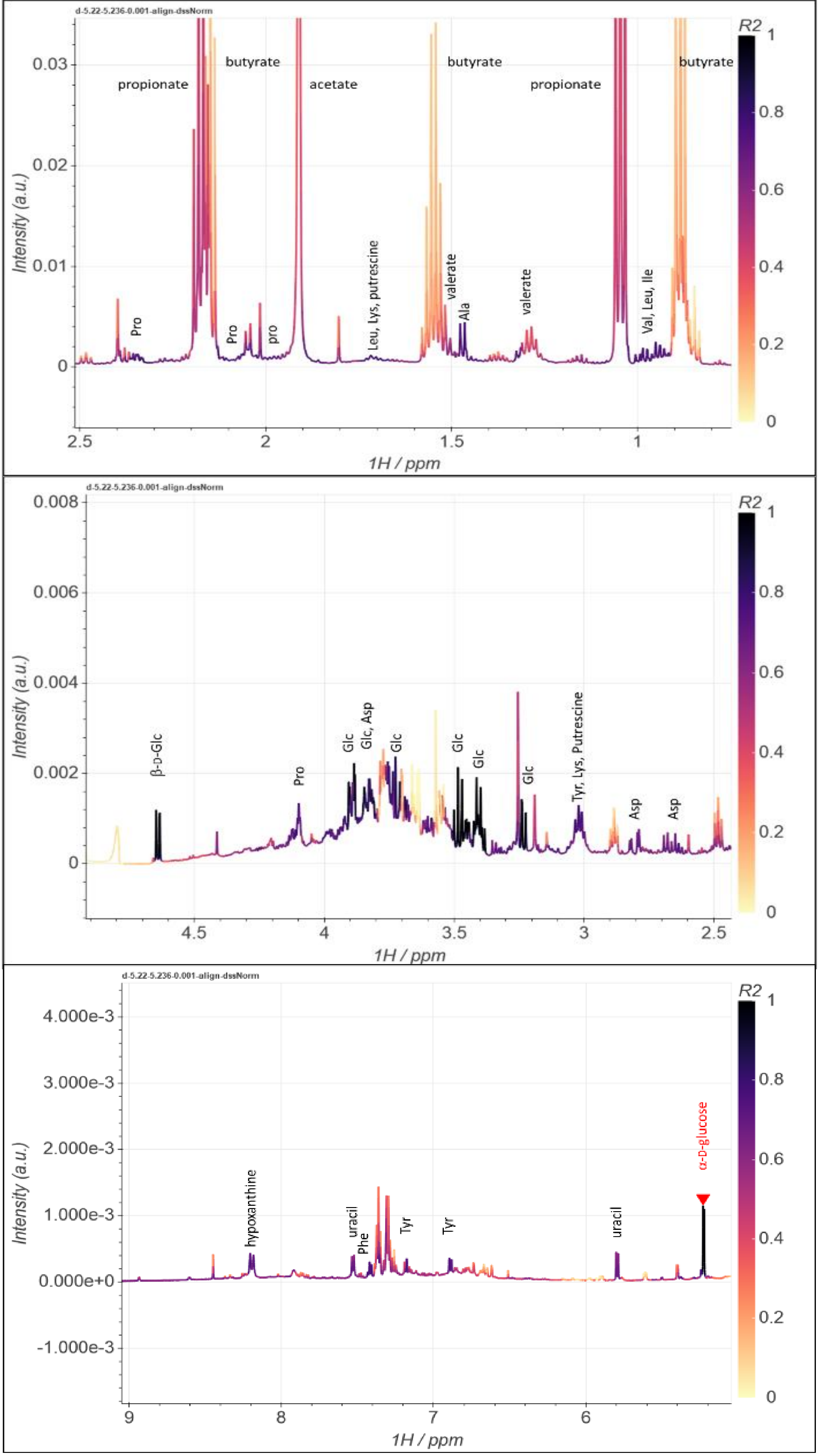

Supplementary Figure 4S. Colour coded median spectrum obtained by using the signal of **glucose, Glc** at 5.23 ppm**,** as the driver signal. The square of the Pearson correlation coefficient, R^2, is coded into the colour of the peaks.

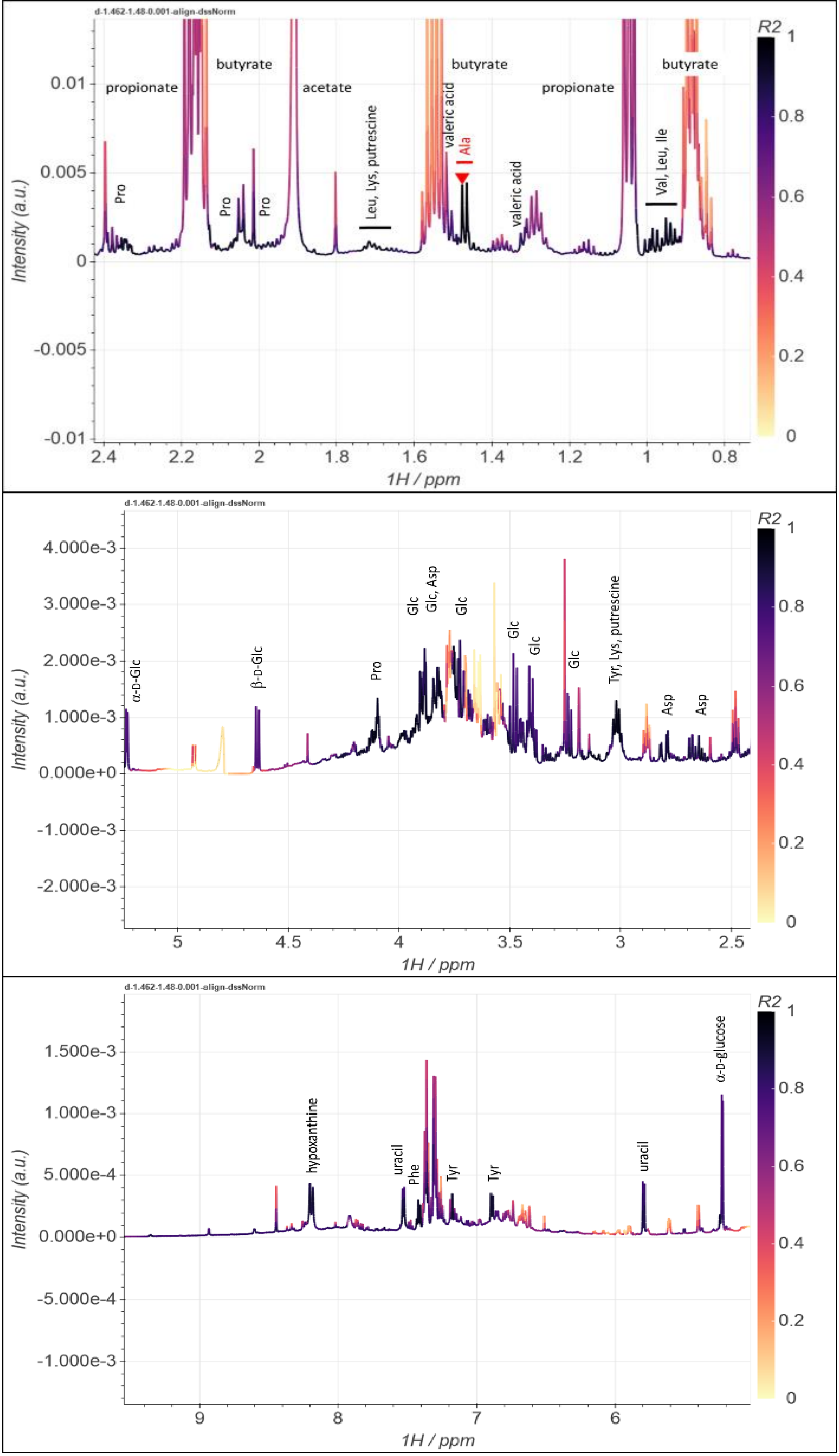

Supplementary Figure 5S. Colour coded median spectrum obtained by using **alanine** at 1.48 ppm**,** as the driver signal. The square of the Pearson correlation coefficient, R^2, is coded into the colour of the peaks.

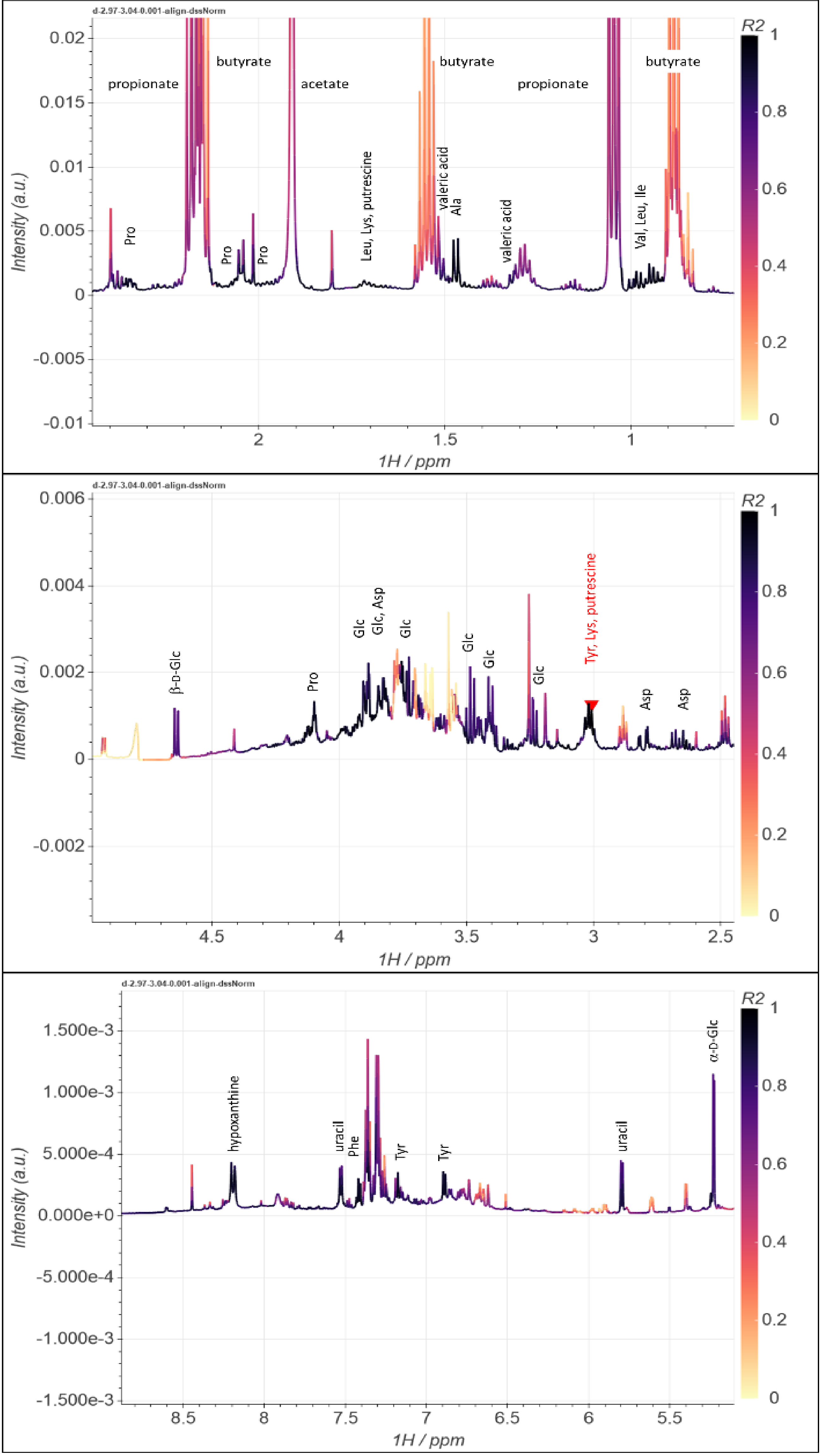

Supplementary Figure 6S. Colour coded median spectrum obtained by using the signal at 3.00 ppm (**Leu, Lys, putrescine**) as the driver signal. The square of the Pearson correlation coefficient, R^2, is coded into the colour of the peaks.

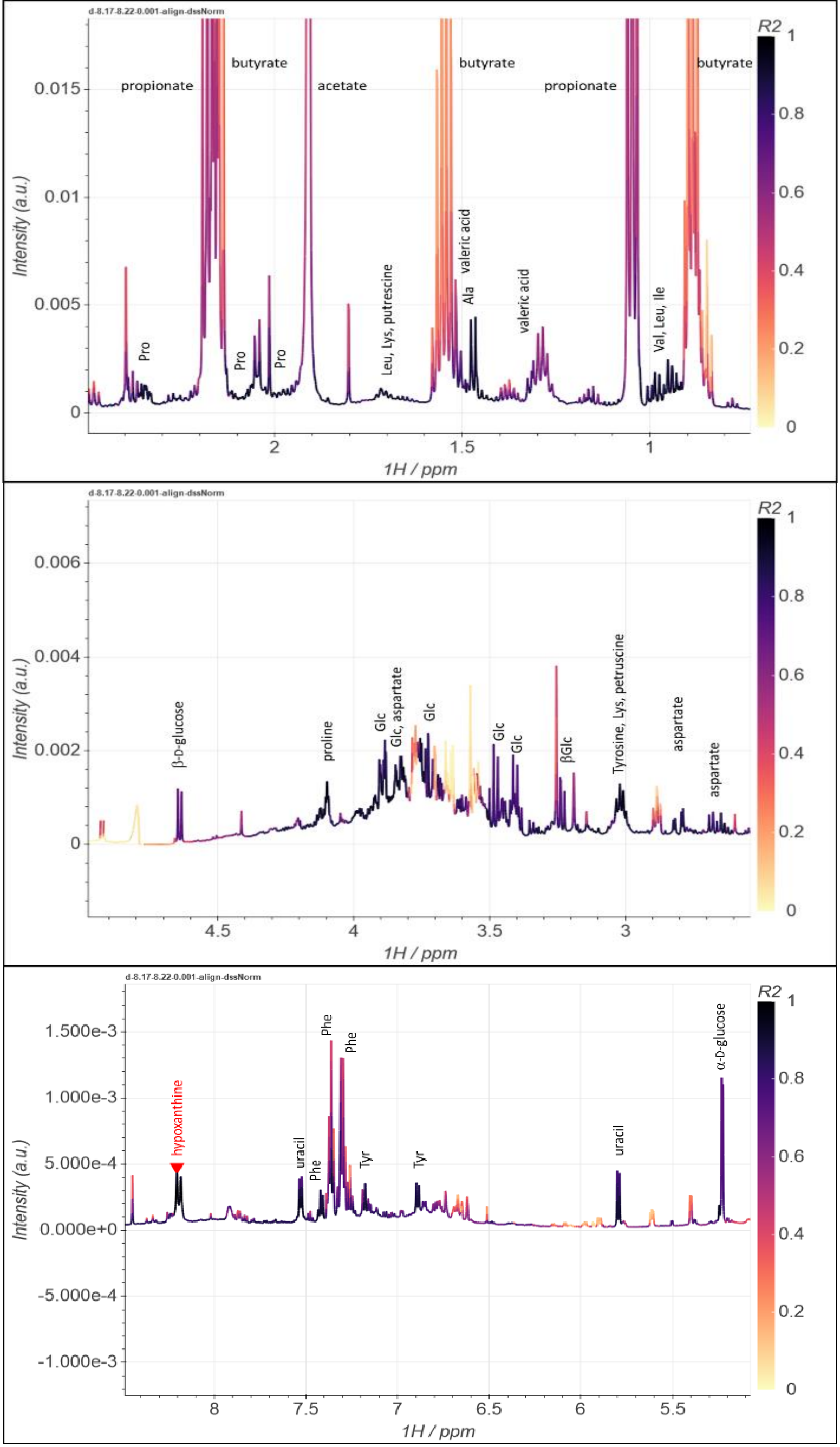

Supplementary Figure 7S. Colour coded median spectrum obtained by using the signals of **hypoxanthine** around 8.20 ppm as the driver signal. The square of the Pearson correlation coefficient, R^2, is coded into the colour of the peaks.

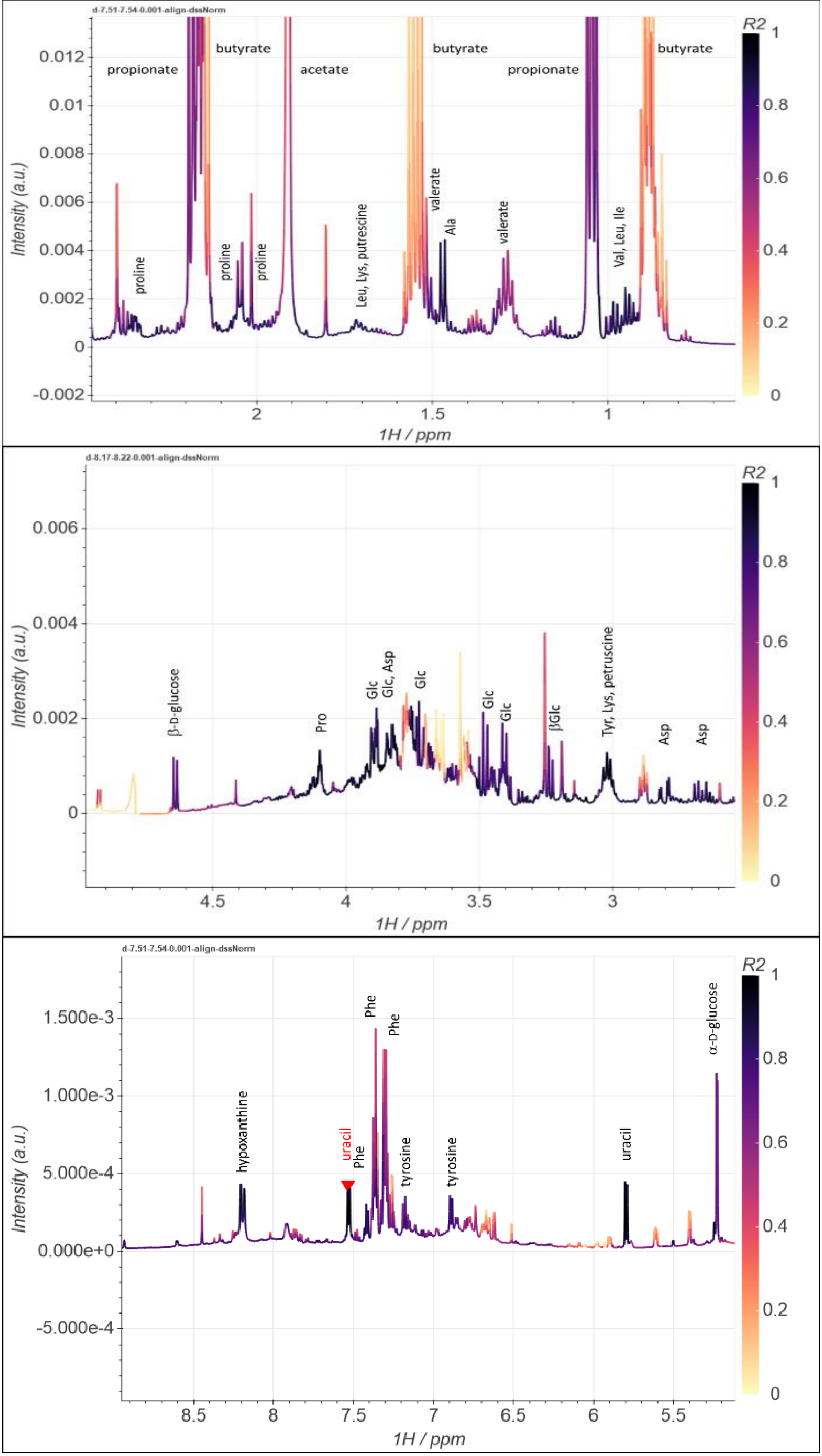

Supplementary Figure 8S. Colour coded median spectrum obtained by using the signal of **uracil** at 7.52 ppm as the driver signal. The square of the Pearson correlation coefficient, R^2, is coded into the colour of the peaks.

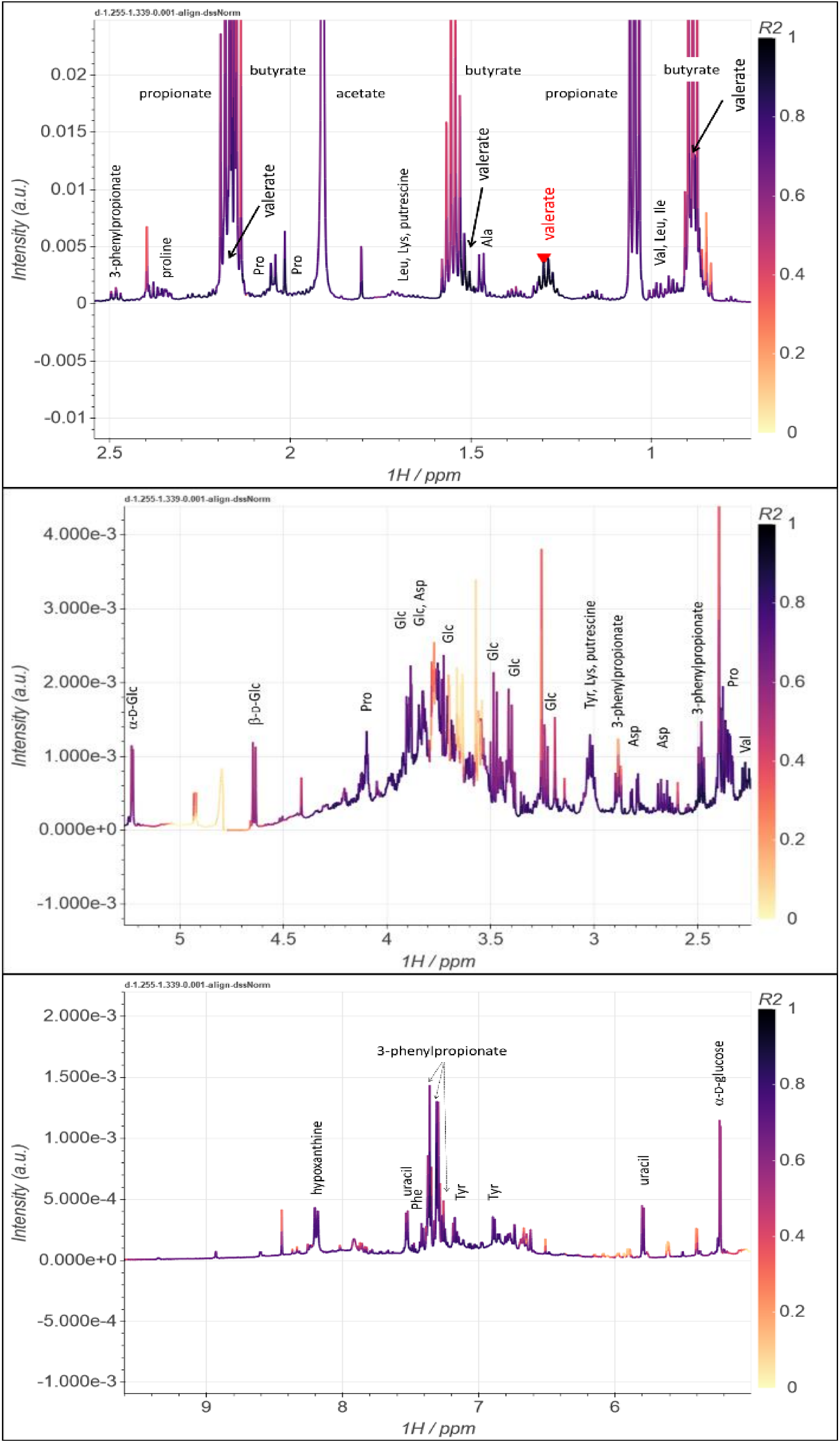

Supplementary Figure 9S. Colour coded median spectrum obtained by using the signal of **valerate** at 1.30 ppm**,** as the driver signal. The square of the Pearson correlation coefficient, R^2, is coded into the colour of the peaks.

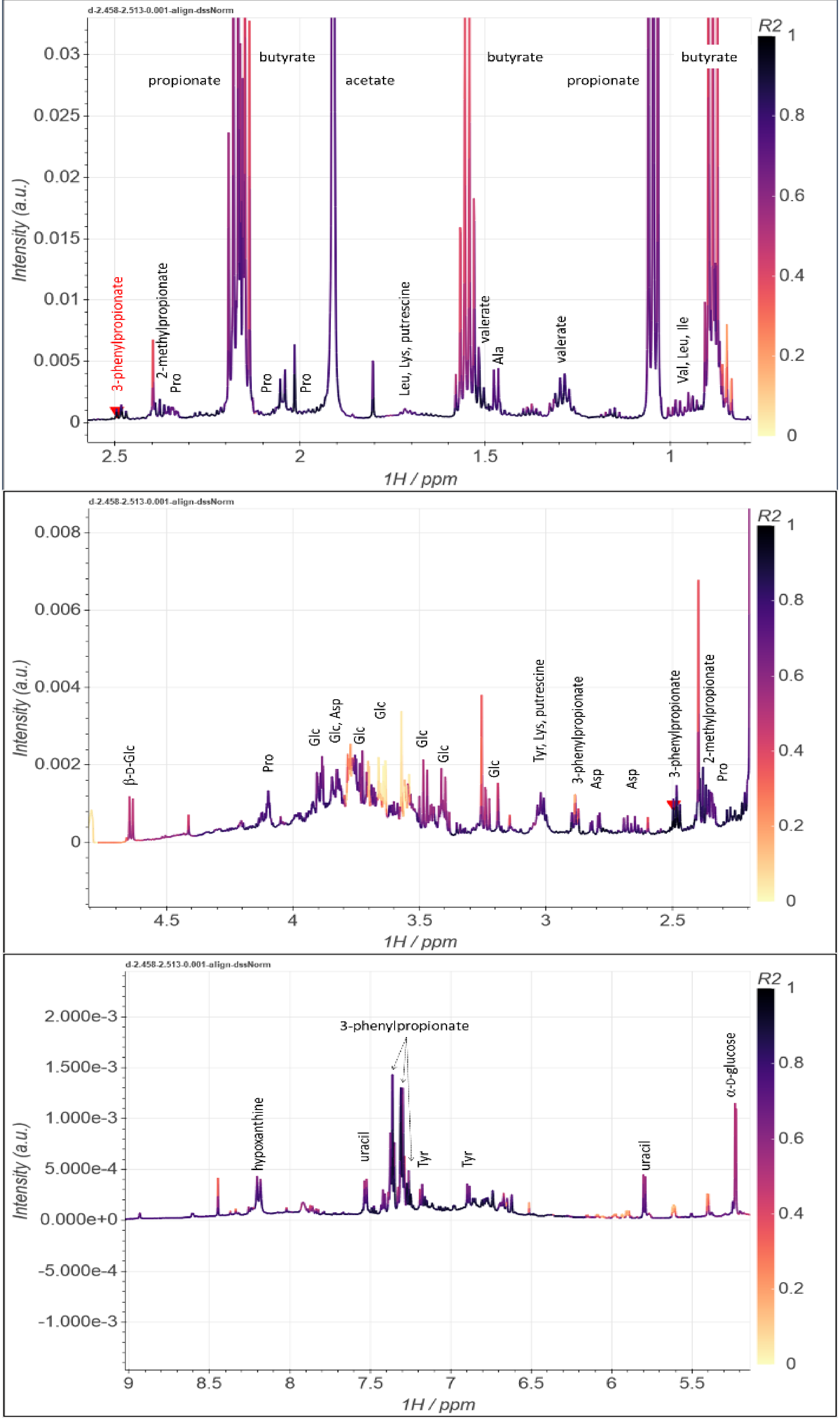

Supplementary Figure 10S. Colour coded median spectrum obtained by using the signal of **3-phenylpropionate** at 2.59 ppm as the driver signal. The square of the Pearson correlation coefficient, R^2, is coded into the colour of the peaks.

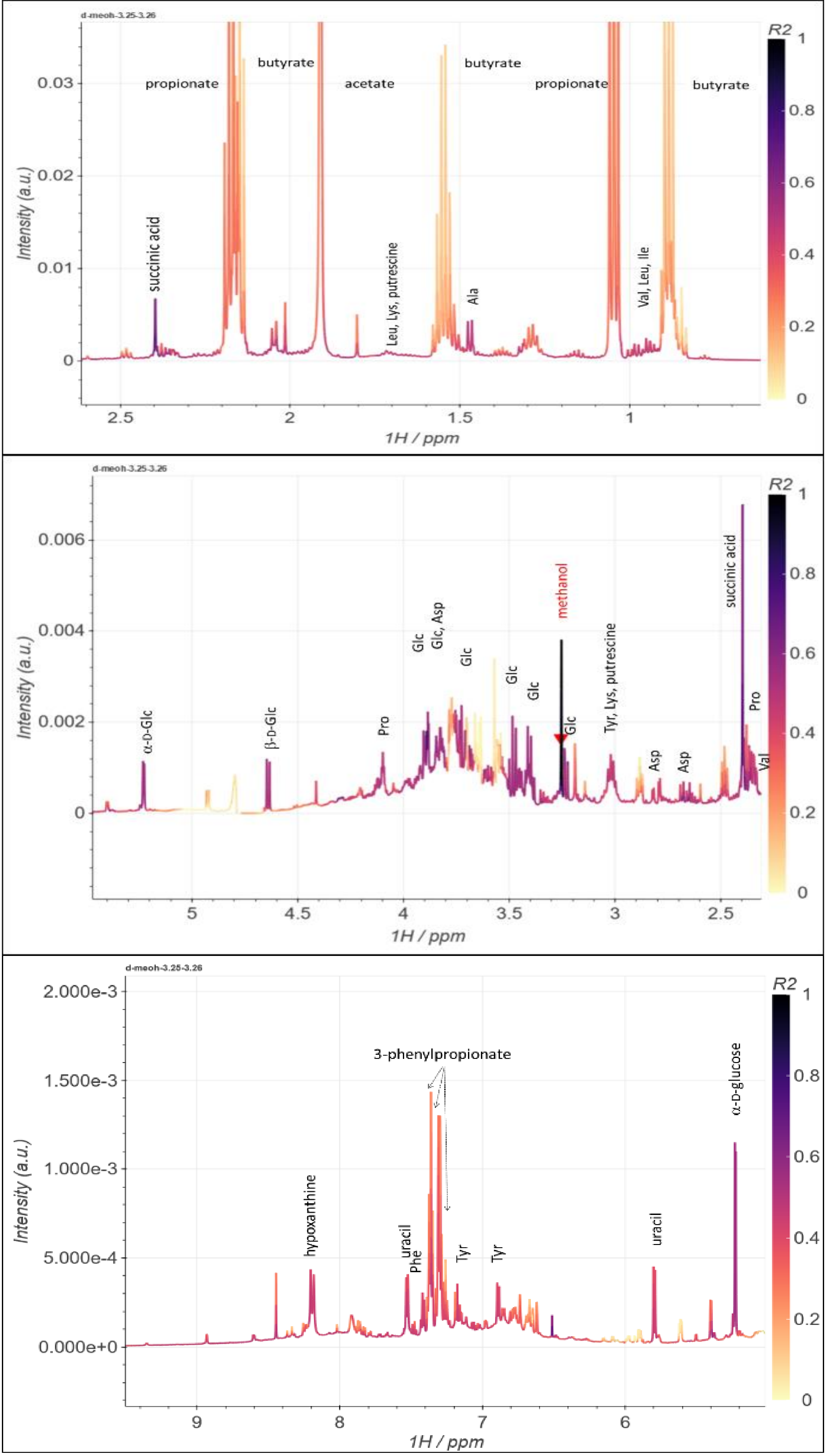

Supplementary Figure 11S. Colour coded median spectrum obtained by using the signal of **Methanol** at 2.59 ppm as the driver signal. The square of the Pearson correlation coefficient, R^2, is coded into the colour of the peaks.

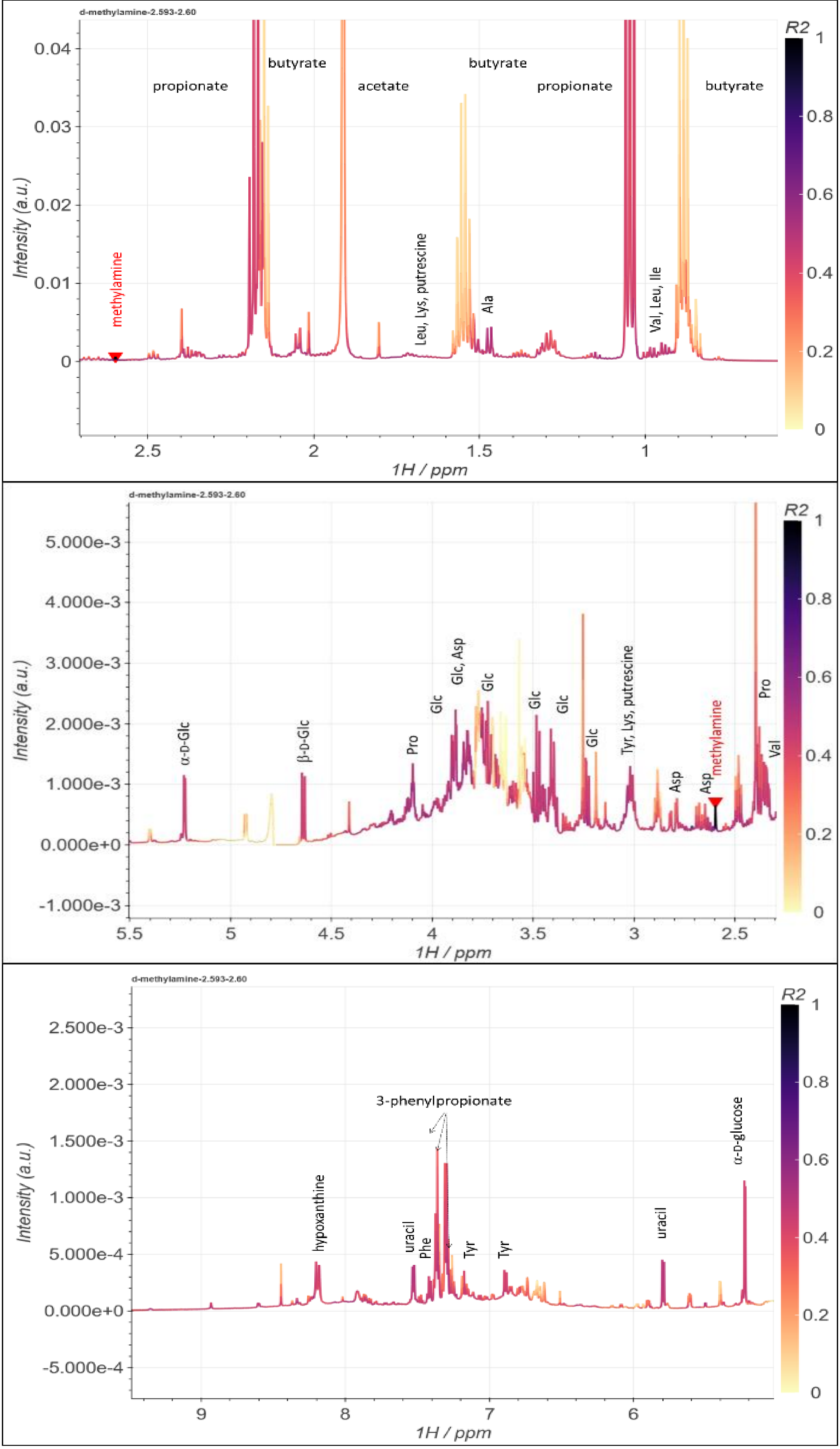

Supplementary Figure 12S. Colour coded median spectrum obtained by using the signal of **Methylamine** at 2.59 ppm as the driver signal. The square of the Pearson correlation coefficient, R^2, is coded into the colour of the peaks.

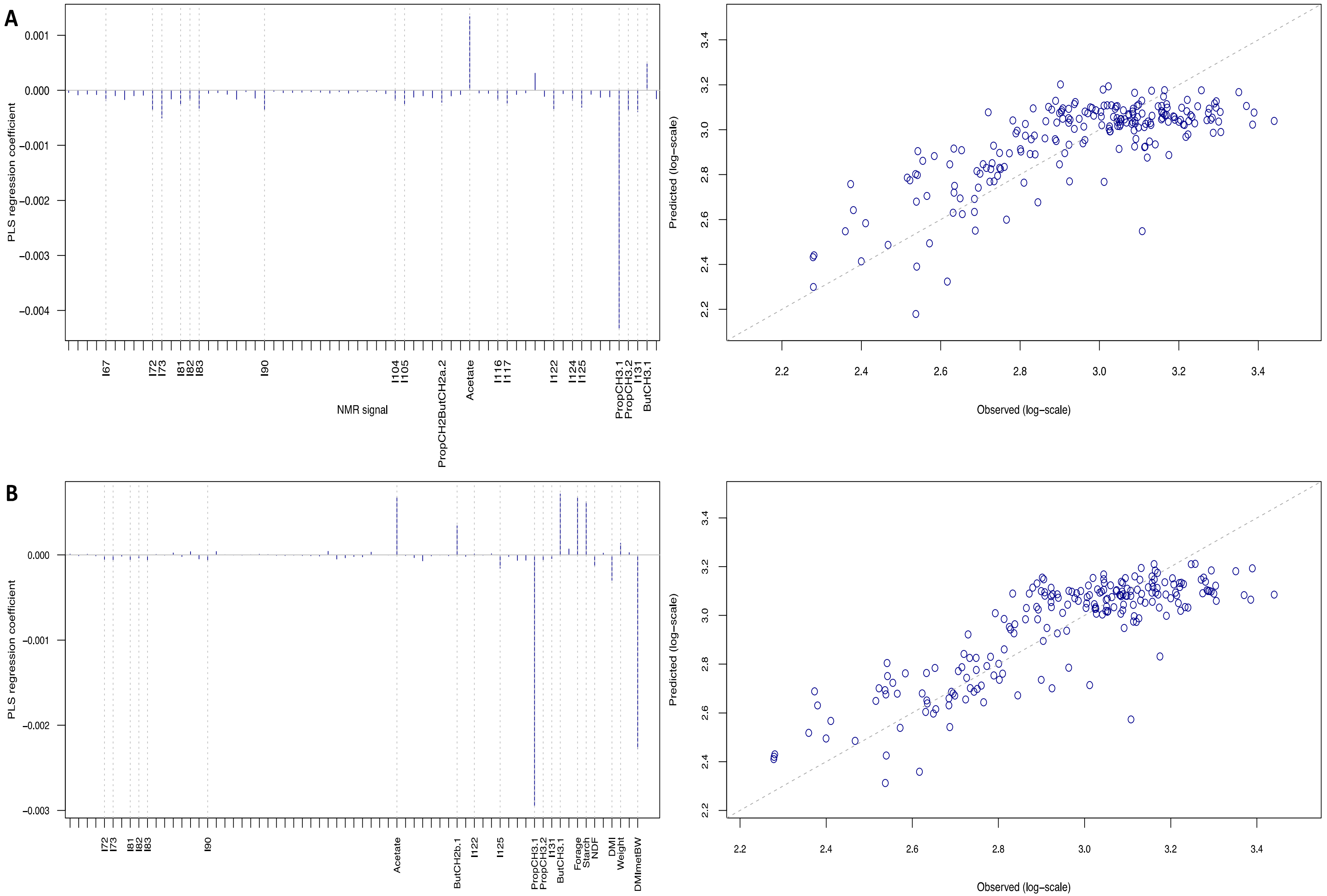

Supplementary Figure 13S. Top (A): PLS regression coefficients (left) and predicted vs observed values (right) for model 1. Bottom (B): PLS regression coefficients (left) and predicted vs observed values (right) for model 2.

Supplementary Table 1S: Metabolites identified by sTOCSY-HSQC and their respective ^{1}H and ^{13}C chemical shifts.

Compound	Structure and ^{1}H and ^{13}C chemical shifts /ppm	Compound	Structure and ^{1}H and ^{13}C chemical shifts /ppm
1. Nicotinate	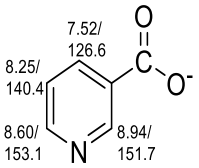	15. 2-Methyl butyrate	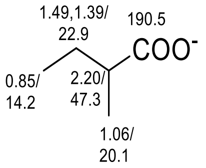
2. Formate	8.46/ 171.9 $HCOO^-$	16. Valine	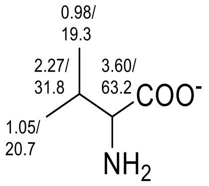
3. Hypoxanthine	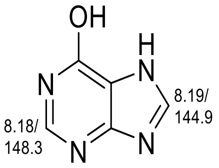	17. Leucine	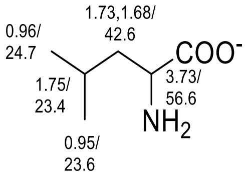
4. Xanthine	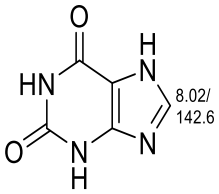	18. Isoleucine	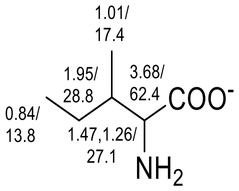
5. Uracil	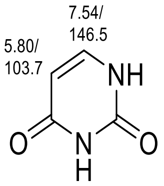	19. Alanine	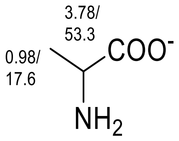
6. Phenylalanine	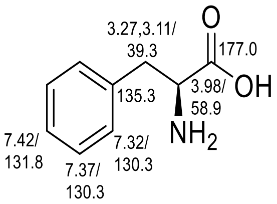	20. Valerate	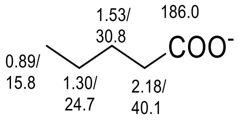
7. 3-Phenylpropionate	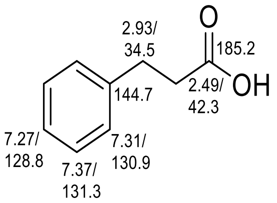	21. Lysine	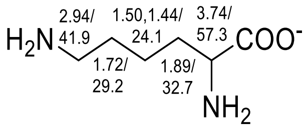
7a. Phenyl acetic acid	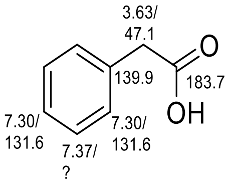	22. Putrescine	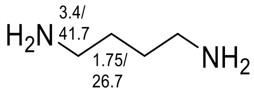
8. Tyrosine	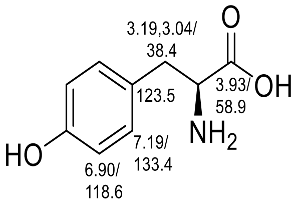	23. Aspartate	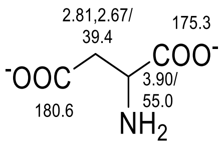

9. α/β-D-glucose	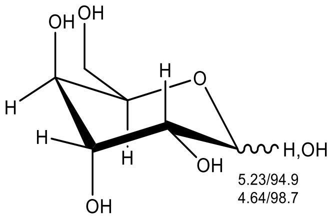	24. Proline	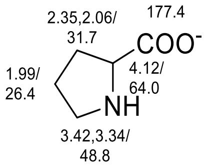
10. Acetate	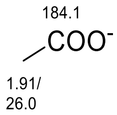	25. Glyceric acid	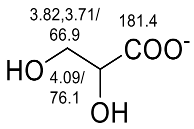
11. Propionate	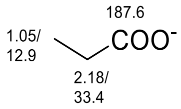	26. 5-oxo-L-proline	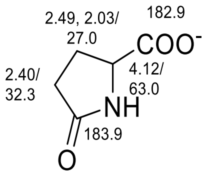
12. Butyrate	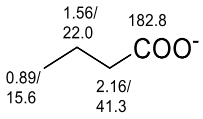	27. Methanol	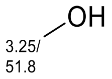
13. 2-methyl propionate (isobutyrate)	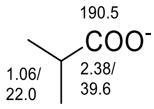	28. Methylamine	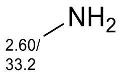
14. 3-Methyl butyrate (isovalerate)	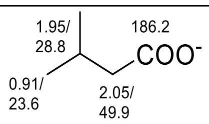	29. Succinic acid	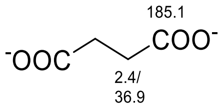
